# Practical aspects of estimating energy components in rodents

**DOI:** 10.3389/fphys.2013.00094

**Published:** 2013-05-01

**Authors:** Jan B. van Klinken, Sjoerd A. A. van den Berg, Ko Willems van Dijk

**Affiliations:** ^1^Department of Human Genetics, Leiden University Medical CenterLeiden, Netherlands; ^2^Laboratory for Clinical Chemistry and Hematology, Amphia HospitalBreda, Netherlands; ^3^Department of General Internal Medicine, Leiden University Medical CenterLeiden, Netherlands

**Keywords:** indirect calorimetry, rodent models, component analysis, resting metabolic rate, activity related energy expenditure

## Abstract

Recently there has been an increasing interest in exploiting computational and statistical techniques for the purpose of component analysis of indirect calorimetry data. Using these methods it becomes possible to dissect daily energy expenditure into its components and to assess the dynamic response of the resting metabolic rate (RMR) to nutritional and pharmacological manipulations. To perform robust component analysis, however, is not straightforward and typically requires the tuning of parameters and the preprocessing of data. Moreover the degree of accuracy that can be attained by these methods depends on the configuration of the system, which must be properly taken into account when setting up experimental studies. Here, we review the methods of Kalman filtering, linear, and penalized spline regression, and minimal energy expenditure estimation in the context of component analysis and discuss their results on high resolution datasets from mice and rats. In addition, we investigate the effect of the sample time, the accuracy of the activity sensor, and the washout time of the chamber on the estimation accuracy. We found that on the high resolution data there was a strong correlation between the results of Kalman filtering and penalized spline (P-spline) regression, except for the activity respiratory quotient (RQ). For low resolution data the basal metabolic rate (BMR) and resting RQ could still be estimated accurately with P-spline regression, having a strong correlation with the high resolution estimate (*R*^2^ > 0.997; sample time of 9 min). In contrast, the thermic effect of food (TEF) and activity related energy expenditure (AEE) were more sensitive to a reduction in the sample rate (*R*^2^ > 0.97). In conclusion, for component analysis on data generated by single channel systems with continuous data acquisition both Kalman filtering and P-spline regression can be used, while for low resolution data from multichannel systems P-spline regression gives more robust results.

## Introduction

In the last two decades, metabolic chambers employing open flow indirect calorimetry have become a standard tool in the study of obesity in humans and rodent models. The time-dependent character of the data generated by such devices contains a wealth of information and provides detailed insights into energy metabolism and changes therein due to physical activity (PA), feeding, and experimental interventions. Using the proper experimental protocol and device settings, it becomes possible to quantify the components that make up total energy expenditure (TEE) and to determine the time response to metabolic challenges (Even and Nadkarni, 2012).

Central to component analysis of indirect calorimetry data that is obtained in freely moving animals or humans is the separation of activity and resting energy expenditure. Several mathematical methods have been proposed for this purpose over the years, amongst which linear regression (Ravussin et al., 1986; Kumahara et al., 2004; Bjursell et al., 2008), Kalman filtering (Even et al., 1991), and penalized spline regression (Van Klinken et al., 2012). The correct application of these methods in the analysis of indirect calorimetry data is challenging and care has to be taken that the derived biological parameters provide an accurate reflection of energy metabolism. Moreover, the performance of these methods depends on several factors such as the sampling frequency of the respiratory exchange, the chamber washout time, and the type of activity sensor. Therefore, understanding of how these mathematical methods work and of how experimental settings affect the data and, in turn, the precision of the energy component estimates is vital for designing optimal experiments and for maximally exploiting indirect calorimetry datasets.

We here discuss the computational procedures that can be used for inferring energy components from time-dependent indirect calorimetry data and we discuss the various factors that affect the precision and performance of these methods. To provide practical and quantitative insight into how each approach works and what their differences are, we analysed high resolution datasets of mice and rats and investigated the effect of several experimental settings.

## Estimation of energy components

### General principles

TEE in animals and man can be subdivided into four main components: the basal metabolic rate (BMR), which is the minimum amount of energy that is needed by the body to sustain vital functions, the activity related energy expenditure (AEE), which is the energy associated with muscular work, the thermic effect of food (TEF), which is the energy associated with the digestion, absorption and storage of food, and the energy expenditure due to thermoregulation (TR), which is the additional heat generated to keep the body at a constant temperature (Blaxter, 1989; Bursztein et al., 1989; Cannon and Nedergaard, 2011).

Employing calorimetric techniques one can have direct access only to the TEE, and experimental interventions and dedicated computational techniques are required to disentangle its components. The set of procedures that is involved with decomposing TEE is referred to as component analysis. For indirect calorimetry data obtained in freely moving subjects, the first step of component analysis consists of separating the activity related energy component from the resting component.

(1) $$\begin{array}{l} \text{TEE}(t)=\text{AEE}(t)+\underset{}{\underset{︸}{\text{BMR}(t)+\text{TEF}(t)+\text{TR}(t)}}\\ \;=\text{AEE}(t)+\text{ RMR}(t) \end{array}$$

The energy component not involved in PA is commonly referred to as the resting metabolic rate (RMR) (Blaxter, 1989; Bursztein et al., 1989), or the background metabolism (Even et al., 1991), and comprises the BMR, TEF, and energy expenditure due to TR. Since each energy component can show large variations in intensity during the course of a day or over longer periods, the time dependence (*t*) is explicitly stated in (1).

**Figure 1:**
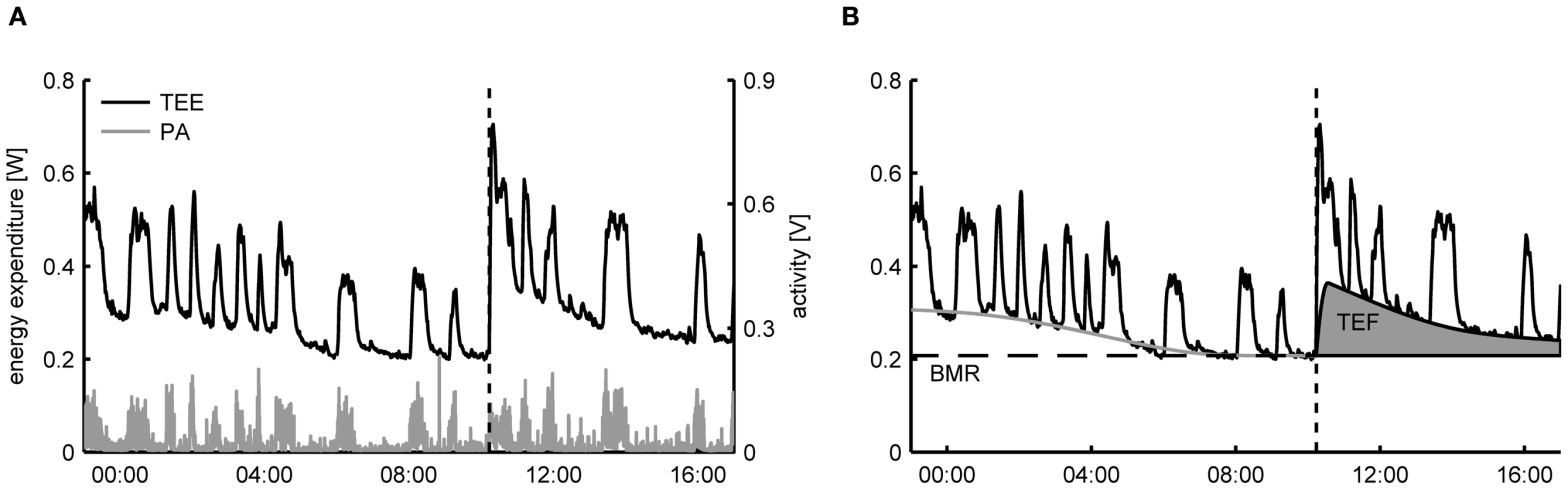
**(A)** Typical dataset of total energy expenditure (TEE) and physical activity (PA) of a mouse measured in a metabolic cage employing open flow indirect calorimetry. From the time patterns in TEE and PA it can be clearly seen that the fast fluctuations in the TEE are correlated with the PA. At 10:00 (dashed line) a single meal was given, after which TEE increased due to the thermic effect of food (TEF). **(B)** Decomposition of TEE consists of exploiting the correlation between the fast time fluctuations in TEE and PA to infer the resting metabolic rate (RMR) and the activity related energy expenditure in a time dynamic fashion. The basal metabolic rate (BMR) is calculated here as the RMR after an overnight fast and the TEF is quantified as the increase in RMR above the BMR after feeding.

For freely moving animals the decomposition of TEE into an activity related and resting component can be achieved by exploiting the time correlation that exists between recorded activity patterns and the TEE. As an example, in Figure 1A the time-dependent TEE and the activity pattern of a mouse are displayed, clearly showing that the fast increases in TEE overlap with the onset of periods of PA. Importantly, since the changes in TEE due to activity are much quicker than the time variation in the other components, it becomes statistically feasible to identify the activity related component in the TEE. More specifically, the basic assumption of TEE decomposition is that—for successive measurements taken in the same subject—the intensity of PA has a strong linear correlation with the AEE

(2) AEE(t)=CCA(t)·PA(t)+e(t)

with CCA the caloric cost of activity, which is the relative amount of energy needed for an activity bout, and *e* the residual energy expenditure, which is assumed to be a normally distributed random variable with zero mean. Since different types of activity can have different caloric costs (Meyer and Guillot, 1986; Heglund and Taylor, 1988), the CCA is time dependent. The CCA is an interesting variable in itself and has been suggested as a measure of the mechanical efficiency of an organism and the coupling between oxygen consumption and ATP production in muscles (Even and Nadkarni, 2012). It is important to note though that the estimate of the CCA also depends on the type of activity sensor, and therefore the validity of biological interpretations of the CCA ultimately depends on the quality of the activity sensor and on how accurately it quantifies the intensity of activity.

The first step in component analysis revolves around estimating the CCA, which permits determining the time-dependent RMR by Equations (1) and (2). Computational techniques for estimating CCA, AEE, and RMR from indirect calorimetry data will be discussed in the next section. The second step of component analysis consists of decomposing RMR further with experimental interventions. The energy component related to TR can be removed by housing animals in thermoneutral conditions, which for mice lie around 30°C and for rats 28°C (Cannon and Nedergaard, 2011; Tschöp et al., 2011; Even and Nadkarni, 2012). Lately, it has been argued that thermoneutrality should be the standard condition for evaluating energy expenditure in rodents rather than at typical ambient temperatures of 20–23°C (Cannon and Nedergaard, 2011; Even and Nadkarni, 2012). The first reason is that under these conditions rodents represent a better model for obesity in humans, since humans normally live under thermoneutral conditions because of clothing. Secondly, below thermoneutral temperatures non-shivering thermogenesis is a variable energy component that can compensate for—and hence mask—variation in other components such as the AEE (Humphries and Careau, 2011; Virtue et al., 2012).

The RMR at thermoneutrality, or RMRt (Speakman et al., 2004), consists of the BMR and TEF. The BMR can be determined by estimating the RMRt after a period of fasting to remove the TEF component. There is some debate on the optimal length of the fasting period to reliably assess BMR in rodents. Some researchers advise a relatively short period of 4–5 h (Speakman, 2013), in order to prevent the animal to go into a state of torpor which decreases the BMR (Hudson and Scott, 1979), while others use a period of overnight fasting (typically more than 12 h) to be sure that the TEF component is completely removed from the RMRt (Even and Nadkarni, 2012). We will abstain here from advocating either approach but simply state that for the communication of experimental results it is important to clearly declare how BMR was measured and how long animals were fasted in order to make the comparison of results from different studies possible.

After a period of fasting the TEF can be determined by presenting a single meal and subsequently calculate the area under the curve of the increase in RMRt above the BMR (Figure 1B) (Even et al., 1994). The same protocol is used for determining TEF in humans, with the exception that energy expenditure is then determined after the consumption of a meal in a resting, supine posture, which makes the correction for activity unnecessary (Reed and Hill, 1996). An alternative approach for determining the TEF that allows subjects to freely move and consume multiple meals during the day is to use regression analysis to simultaneously estimate AEE and TEF from the time variations in the TEE (Van Milgen et al., 1997; Van Milgen and Noblet, 2000). However, since the dynamic response of energy expenditure on food intake is much slower than PA, statistically it is only possible to discern TEF from the RMR using regression if meals are consumed separated by large enough time intervals—typically around three meals per day—such that sufficient time variation in the TEE is caused by food intake. As a consequence, this approach cannot be applied to rodents that are given *ad libitum* access to food, as then moments of food intake will occur with a high frequency (Moran, 2003).

It is important to note that except for a transient increase in energy expenditure, food intake can also have additional effects on energy metabolism. For instance, as was shown by Feldmann et al. (2009), adrenergic thermogenesis is increased in wild type mice when put on a high fat diet under thermoneutral conditions. Disentangling the effect of diet composition on energy expenditure from the TEF may be difficult in data from single animals because the two processes will overlap in time. Rather, the effects of diet composition must be assessed by comparing energy components between groups of mice that have been put on different diets.

An interesting extension of component analysis is to decompose the time-dependent oxygen consumption and carbon dioxide production separately (Van Milgen et al., 1997). In this way it becomes possible to calculate the respiratory quotient (RQ) related to activity and resting metabolism, which permits to investigate fuel selection in greater detail. For instance, from the dynamic response of the activity and resting RQ after food intake or other metabolic challenges, insight can be gained into the regulation of substrate oxidation and metabolic flexibility (Kelley and Mandarino, 2000; Even and Nadkarni, 2012).

## Methods

### Alignment of activity and energy expenditure data

Over the years several computational methods have been proposed for estimating the activity related part of TEE. These methods are based on assumption (2), namely on that there exists a strong correlation between the time patterns of AEE and PA. However, the correlation between the raw time sequences of the PA and energy expenditure is usually very poor and preprocessing of the data is needed to maximize their correlation and make TEE decomposition possible. The most important step in data preprocessing is to take into account the fact that the time pattern in the respiratory exchange is dampened due to gas mixing in the metabolic chamber, while activity measurements are instantaneous (Arch et al., 2006; Lighton, 2008). Modeling the chamber as a linear compartment, the effect of gas mixing is mathematically described by the impulse response function *h(t)*

(3) $$h(t)=\{\begin{array}{cc} \frac{1}{\tau }e^{-\frac{t-\tau_{\text{delay}}}{\tau }} & t\geq \tau_{\text{delay}}\\ 0 & t<\tau_{\text{delay}} \end{array}$$

with τ the washout time of the chamber, which in the case of ideal mixing is equal to the chamber volume divided by the air flow, and τ_delay_ the delay introduced by the tubing and gas dryers that are situated between the outlet of the chamber and the gas sensors. In practice τ may be found 5–10% lower than its theoretic value—that is, the ratio of the chamber volume and air flow—because of dead spaces in the chamber. A more precise model of gas diffusion also takes into account the gas diffusion inside the body, which extends (3) to a two compartment model; for details, see Van Klinken et al. (2012). However, since the washout time induced by the chamber is typically much larger than that of the animal (often by a factor of least 10) the single compartment model normally gives a reasonable approximation.

Given τ and τ_delay_, there are two possible solutions to align the raw PA and TEE time sequences. The first approach consists of applying the impulse response *h(t)* on the activity data and thus induce the same deformation as on the gas exchange. This approach is computationally the simplest and involves the application of a so-called infinite impulse response filter to the activity data. For a single compartment model and a sequence of activity data PA(*i*) measured with sample time *T*, the diffusion corrected activity PA^*^(*i*) is calculated as

(4) $${\text{PA}}^{*}(i)=a\cdot {\text{PA}}^{*}(i-1)+b\cdot \text{PA}(i)$$

with *i* the index in the PA sequence and *a* and *b* the filter coefficients derived from the impulse invariance method (Oppenheim et al., 1999)

$$a=\exp\left(-\frac{T}{\tau }\right)\;b=1-\exp\left(-\frac{T}{\tau }\right)$$

where τ and *T* are expressed in the same time units. In addition, a linear shift must be applied to account for τ_delay_. This approach works well for both high and low resolution data, but requires the washout time τ to be relatively small such that the fast variations in TEE due to activity are not dampened to the extent that the correlation between TEE and PA is lost. Alternatively, if τ is large, as is the case for human metabolic chambers or those for large mammals, the instantaneous TEE needs to be calculated, which is a mathematical procedure called deconvolution (Arch et al., 2006; Lighton, 2008; Tokuyama et al., 2009). Deconvolution of the TEE time series, though, is a more complicated procedure because it is very susceptible to sensor noise and therefore requires the application of filtering techniques to attenuate the effect of noise. Consequently, deconvolution can only be applied on frequently sampled data, as this increases the ability of filters to get rid of sensor noise while leaving the original signal intact.

### Linearisation of activity and energy expenditure data

In addition to the correction for gas mixing effects, it may sometimes be necessary to apply a non-linear transformation to the raw activity data in order to linearize the relation between activity and energy expenditure. Other mathematical functions that can be applied to the activity signal are a threshold to correct for a baseline activity signal or a kernel to smoothen noisy activity data; for details, see Van Klinken et al. (2012), supplemental text 2. Whether or not there is a need to transform activity data can be investigated by inspecting the scatter plot of the diffusion corrected activity and the TEE or the residuals from component analysis. If non-linear trends are perceivable in these plots then an appropriate function must be chosen to transform the activity data, since otherwise the linearity assumption (2) is violated and decomposition may become unstable. The best practice for selecting the free parameters of the non-linear transformation is to minimize the residual variation that results from the decomposition method. Since the same transformation must be applied to the activity data from all subjects participating in an experiment, it is important to minimize the sum of residuals from all datasets.

**Figure 2:**
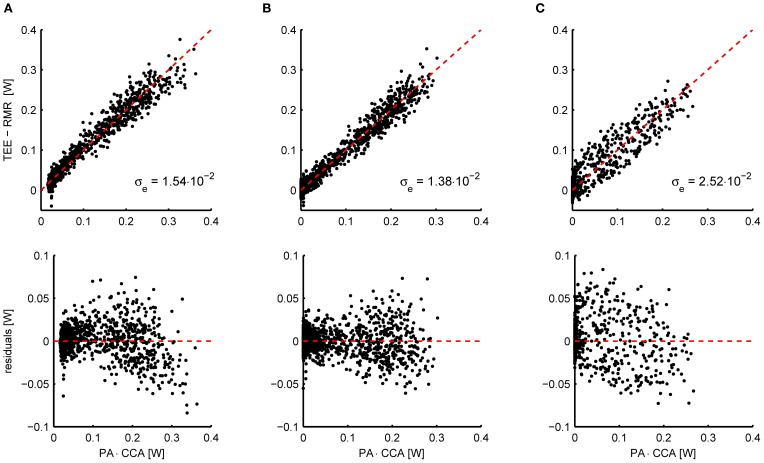
**(A)** Scatterplot of the activity signal from piezo-electric sensors corrected for gas mixing and the total energy expenditure (TEE) minus the resting metabolic rate (RMR) in mice (*n* = 11) (top) and the residual energy expenditure TEE – RMR – AEE (bottom). The AEE and RMR were calculated with P-spline regression. The relation between energy expenditure and activity is slightly curved, which is accentuated when plotting the residuals of the fitted P-spline model against the activity. Moreover, a non-zero baseline is present in the activity signal. **(B)** Applying a threshold to correct for the baseline activity signal and an exponent of 0.6, the relation between activity and energy expenditure is sufficiently linearized. As a result of the preprocessing, the standard deviation in the residuals is reduced from σ_e_ = 1.54 × 10^−2^ W to σ_*e*_ = 1.38 × 10^−2^ W. The preprocessing parameters were fitted by minimizing the residuals of the P-spline model summed over all mice. **(C)** Instead of applying the linear compartment model to the PA signal to increase the correlation between the TEE and PA time series, they can also be binned in a given time interval (here 15 min). Although this procedure increases the correlation between both signals with respect to the untransformed data, binning does give inferior results when compared to the linear compartment model **(B)**.

As an example, Figure 2A shows the relation between the energy expenditure and the activity signal from piezo-electric sensors measured in 11 mice. To properly overlap the data of all mice, the activity (*x*-axis) has been multiplied by the subject specific CCA that resulted from penalized spline (P-spline) regression. The scatter plot shows that there exists a slight non-linear relation between the activity and the energy expenditure, which is confirmed by the scatter plot of the residual energy expenditure and the activity. Moreover, the activity signal contains a non-zero baseline, which needs to be subtracted from the total activity signal to prevent the AEE estimate from being biased. Applying a threshold and an exponent of 0.6 linearizes the relation and shifts the left tail of the point cloud to the origin (Figure 2B).

An alternative approach for aligning TEE and PA data is to bin both signals into intervals of a given length; e.g., see Nonogaki et al. (2003), Bjursell et al. (2008), and Virtue et al. (2012). Technically this procedure corresponds to the application of a low pass filter to both signals, which makes sense because the low frequency TEE and PA signals correlate better in time. Nevertheless, the resulting correlation is less strong as when applying the linear compartment model, as is illustrated in Figure 2C. Consequently, using the linear compartment model (i.e., Equation 4) should be the preferred approach for aligning TEE and PA data for the purpose of regression analysis, since this gives a smaller residual error and therefore more accurate regression estimates.

### Regression analysis

The classical technique for estimating CCA and decomposing TEE into an activity and resting related part is by performing linear regression of TEE against PA for each subject separately (Ravussin et al., 1986; Nonogaki et al., 2003; Kumahara et al., 2004; Bjursell et al., 2008). Using this approach, the intercept of the regression line with the *y*-axis corresponds to the average RMR and the slope to the CCA. The advantage of this method is that it is relatively simple to use and can be executed by standard (spreadsheet) software packages. However, one of the main disadvantages is that it does not take into account the time variation that is present in the resting energy expenditure, which increases the amount of uncertainty in the CCA and RMR estimates and can introduce a bias into the results.

As was first put forward by Brown and colleagues (1991), it is possible to take into account the time variation of the RMR by modeling the RMR with a more complex, time-dependent function. Recently we proposed a solution for regression based component analysis in which the time-dependent RMR is modeled with P-spline functions (Van Klinken et al., 2012). This makes it possible to obtain an estimate of the time variation in the RMR, but also increases the accuracy with which the CCA and AEE are estimated. In addition, this method has demonstrated to be relatively robust to low sample frequencies and noisy activity measurements, which is important when performing component analysis on data generated by multiplexed systems.

When using P-spline regression for component analysis, first the number of knots has to be chosen in the spline function that models the time variation in the RMR. This parameter determines how quickly the RMR can vary with time. In our earlier study we showed that with approximately 2*k* knots per day, frequency components in the RMR of up to *k* day^−1^ can be estimated (Van Klinken et al., 2012). Importantly, slow time variations in the RMR can be estimated with a higher robustness than fast time variations, but they will also introduce a bias when RMR changes quickly. In practice, inspection of the time-dependent plots of the TEE, PA and fitted RMR is needed for determining the right amount of knots.

### Kalman filtering

An alternative approach to regression analysis is to decompose TEE using Kalman filtering, as was originally proposed by Even and Nicolaidis (1984) and Even et al. (1991). The basic assumption of this method is that the RMR and CCA can be modeled as Gaussian random processes that vary in time. The relation between the RMR, CCA, AEE, and TEE and the effects of gas mixing are expressed in a state space model, which permits the estimation of the state variables by means of Kalman filtering. Kalman filtering is a numerical filtering technique that works by continuously predicting the future state of the system and then correcting the prediction with the measured data. This procedure relies on the fact that data has been sampled with a relatively high frequency, and it is therefore best suited for analysing data generated by single channel indirect calorimetry systems with continuous data acquisition (Even and Nadkarni, 2012). Since the state space model already includes the effects of gas mixing on the measured respiratory exchange, the data must not be corrected for these effects as part of preprocessing. Importantly, the performance of the Kalman filter depends on the choice of filter parameters, which must be set in advance by the user by tuning the filter.

### Minimal metabolic rate

A popular approach for estimating the BMR from indirect calorimetry datasets in animals is to take the minimum energy expenditure that is reached after a period of fasting (Selman et al., 2001; Speakman, 2013). Since random fluctuations in the resting energy expenditure can induce a downward bias in the BMR estimate obtained in this way, normally the minimal energy expenditure is taken averaged over a short, e.g., 5 min, interval. The advantage of this method is that it does not involve correlating PA and TEE—activity does not even have to be measured. A potential downside, however, is that the BMR estimate is influenced by several experimental settings. Most importantly, the registered minimum metabolic rate depends on the duration of the interval over which is sampled and on the sampling frequency, since sampling for a longer period or with a higher frequency will increase the likelihood of finding a low measurement by chance (Cooper and Withers, 2010). Also, the washout time of the metabolic chamber influences the BMR estimate, since if the measured respiratory exchange needs too long to return to base level after an activity bout has occurred then the BMR estimate will be biased upward. Finally, the measured minimum metabolic rate depends on the activity pattern of the animal, since animals that rest more often or have longer pauses will exhibit more minima.

## Comparison of methods

### Experimental data

To make a baseline comparison between the results that are obtained by decomposition methods, we tested Kalman filtering and P-spline regression on high resolution datasets of mice (*n* = 11) and rats (*n* = 47) that have been made available by P. C. Even. In short, for each animal respiratory exchange was measured during 24 h with a sample time of either 2 or 5 s. Mice were housed at 30°C and rats at 28°C under a standard 12 h:12 h light/dark cycle. Activity was measured with piezo-electric force transducers and was averaged over periods equal to the sample time. Animals were placed in the metabolic chamber at 18:00 with water but no food and were fasted overnight, after which they were fed a single meal between 9:00 and 10:00. Weir's equation was used to calculate energy expenditure from oxygen consumption and carbon dioxide production rates (Weir, 1949).

We performed P-spline regression to estimate the time-dependent activity related and resting respiratory exchange using a spline function containing 8 knots. Also the CCA was allowed to vary in time and was modeled using a spline function with 16 knots. Because the meal induced a very rapid rise in the energy expenditure, we applied P-spline regression separately on the pre-feeding data and the data obtained 20 min after food intake; the intermediate 20 min interval was interpolated using a 2nd order polynomial function. To increase robustness in the RMR estimate, weighed regression was used where measurements were given a weight of 0.2 during periods of activity and a weight of 1.0 during resting periods. The smoothing parameter λ was estimated automatically using the generalized cross validation criterion as previously reported (Van Klinken et al., 2012) and was subsequently averaged separately for mice and rats.

Kalman filtering was performed on the data under two different conditions: with the filter parameters fitted to minimize the difference between the time-dependent RMR estimates of the Kalman filter and the P-spline model, and with the filter parameters manually tuned without taking the results of the P-spline model into account. The initial estimate of the state vector was obtained by linear regression; the initial 4 h of data were used for the Kalman filter to converge and were not used in the further analysis. To account for the rapid change in energy metabolism after feeding, the process noise variance associated with the CCA was increased by a factor of 10 for a window of 120 min after feeding. For both the Kalman filter and the P-spline regression approach a threshold and non-linear transform was applied to the raw activity data as explained in Figure 2. All calculations were performed in MATLAB (The MathWorks).

### Comparison of the time-dependent RMR

**Figure 3:**
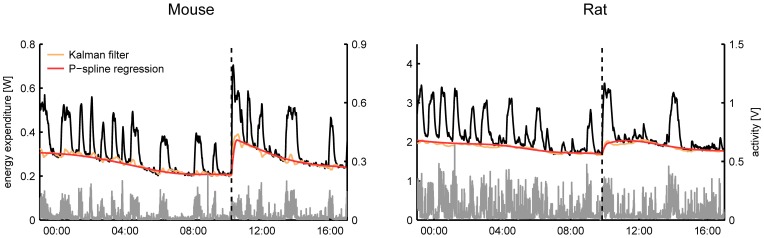
**Time plots of the total energy expenditure, activity and estimated RMR of a mouse and a rat using P-spline regression and Kalman filtering with manually tuned parameters.** On average there is a good correspondence between the results from both methods, with the Kalman filter showing slightly more variation in the RMR estimate than the P-spline method.

From a visual comparison of the time-dependent RMR estimates it followed that even in the case that the Kalman filter was tuned manually there was a good consensus between both methods for most subjects (Figure 3). A general difference that we observed was that the RMR estimated by the Kalman filter exhibited more variation than the result of P-spline regression, especially during periods of activity. This divergence can be explained by the way how both methods operate: the Kalman filter assumes that there is a small residual signal and attributes fast fluctuations to the RMR whereas the P-spline regression model assumes that the RMR varies slowly in time and attributes most of the fast fluctuations to the residuals.

Some care has to be taken when interpreting the fast, non-activity related, time fluctuations in the respiratory exchange as they can have both biological and technical origins. Fast fluctuations of a biological origin are those associated with the sudden increases in energy expenditure due to (endogenous) nervous and hormonal changes. In contrast, variations that are characterized by a negative spike and a subsequent more blunted positive peak are indicative of short periods of apnea and therefore reflect respiratory dynamics rather than true changes in energy expenditure (Speakman, 2013). Importantly, fluctuations observed in the non-activity energy expenditure during activity bouts are often caused by the non-perfect match between the registered activity and the energy expenditure—e.g., due to noise in the activity sensor or to the non-ideal mixing of air in the metabolic chamber—and can therefore be technical in nature.

### Comparison of inferred metabolic parameters

In addition to comparing the time-dependent results we investigated how well the estimates of a number of metabolic parameters corresponded between the Kalman filter and P-spline regression. In detail, we derived the following metabolic parameters from the time-dependent activity and resting VO_2_ and VCO_2_ of each method: the BMR, calculated as the average RMR over a period of 90 min prior to feeding; the TEF, calculated as the area under the curve in RMR above the BMR after feeding for a period of 5 h; the AEE and CCA during the fasting period; the resting (activity) RQ during fasting, calculated as the total resting (activity) VCO_2_ produced divided by the total resting (activity) VO_2_ consumed during a 5 h period prior to feeding; and the increase in resting (activity) RQ due to feeding, calculated as the total resting (activity) VCO_2_ produced divided by the total resting (activity) VO_2_ used during a 5 h period after feeding, *minus* the fasting resting (activity) RQ.

**Figure 4:**
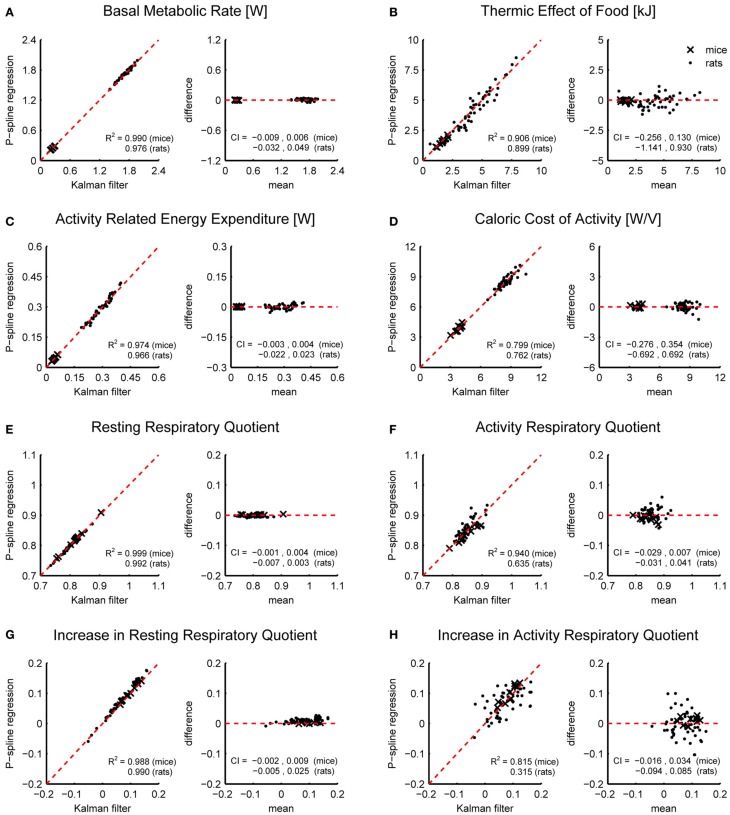
**Correspondence of metabolic parameter estimates from indirect calorimetry datasets of mice and rats using Kalman filtering and P-spline regression.** Bland Altman plots are shown of the BMR **(A)**, TEF **(B)**, AEE **(C)**, CCA **(D)**, resting RQ during fasting **(E)**, activity RQ during fasting **(F)**, increase in resting RQ after feeding **(G)**, increase in activity RQ after feeding **(H)**. The coefficient of determination (*R*^2^) and 95% confidence interval (CI) are shown separately for mice and rats.

Figure 4 shows Bland Altman plots for each of the eight metabolic parameters that were estimated from the results of P-spline regression and Kalman filtering with parameters fitted to minimize the difference with the time-dependent RMR of the P-spline model. It follows that there was a very high correlation between both methods for most metabolic measures, which shows that both methods can give virtually identical results on high resolution data as long as the parameters of the Kalman filter are tuned by using an external criterion. When the filter parameters were tuned manually the correlations generally dropped (Table 1), but were still very good for the BMR, resting RQ during fasting and the increase in resting RQ, with *R*^2^ > 0.95 for both mice and rats. There was also a high agreement between the AEE estimates with *R*^2^ around 0.97, but this dropped to *R*^2^ = 0.91 for rats when manually tuning the filter. The correlation was also strong for the TEF with *R*^2^ between 0.87 and 0.91, while for the CCA the correlation was considerably lower with *R*^2^ between 0.59 and 0.80. The reason why the *R*^2^ of the CCA is much lower than that of the AEE is that the CCA varies less between animals than the AEE: the coefficient of variation of the CCA is 0.09 in mice and 0.08 in rats whereas for the AEE it is 0.27 in mice and 0.20 in rats. Consequently, the denominator of the fraction of unexplained variance is relatively smaller in the CCA, which explains why the *R*^2^ is lower. The correlation of the activity related RQ during fasting and the increase after feeding in mice dropped considerably when manually tuning the filter, which shows that this measure is very sensitive to the choice of filter parameters. For rats the correlation was worse than for mice, which was probably caused by the fact that rats were overall much less active after prolonged fasting and after refeeding, inducing thus a larger degree of uncertainty in the activity RQ estimates.

**Table 1:** **The coefficient of determination (*R*^2^) of the correlation for metabolic measures based on the results of P-spline regression model and of the Kalman filter with automatically and manually tuned filter parameters**.

	**Mice**		**Rats**	
	**Automatic**	**Manual**	**Automatic**	**Manual**
BMR	0.990	0.987	0.976	0.958
TEF	0.906	0.911	0.899	0.866
AEE	0.974	0.975	0.966	0.908
CCA	0.799	0.777	0.762	0.589
Resting RQ	0.999	0.995	0.992	0.978
Activity RQ	0.940	0.878	0.635	0.372
Δ Resting RQ	0.988	0.962	0.990	0.987
Δ Activity RQ	0.815	0.373	0.315	0.188

Automatically tuned parameters of the Kalman filter were fitted as to minimize the difference with the time-dependent RMR estimate of the P-spline regression model, resulting therefore in energy component estimates that are as close to the result of the P-spline regression model as possible. In contrast, manually tuned parameters were based on visual criteria and did not include prior knowledge of the results of P-spline regression. The agreement between metabolic measure estimates is typically worse when the Kalman filter is manually tuned, though correspondence is still good for the BMR, TEF, AEE, and the resting RQ. For the CCA the correlation is less strong, while for the activity RQ during fasting in rats and the increase in activity RQ after feeding the correlation is very poor, showing that these measures are very sensitive to the specific method that is used and to how the parameters were tuned.

The results in Figure 4 show that on high resolution data there is an overall good agreement between the metabolic parameters as determined from the decomposition results of the Kalman filter and P-spline regression method, except for the activity related RQ in rats. The differences between the metabolic parameter estimates are due to the different assumptions that are at the base of both methods, most notably regarding how the time variation in the RMR and CCA is modeled: the Kalman filter uses random Gaussian processes, which exhibit very fast changes, while the P-spline method models the RMR and CCA with spline functions that vary slowly in time. This difference is magnified during periods of activity (Figure 3), which explains why the metabolic measures related to resting metabolism are more accurate than those related to activity.

### Measurement of BMR

**Figure 5:**
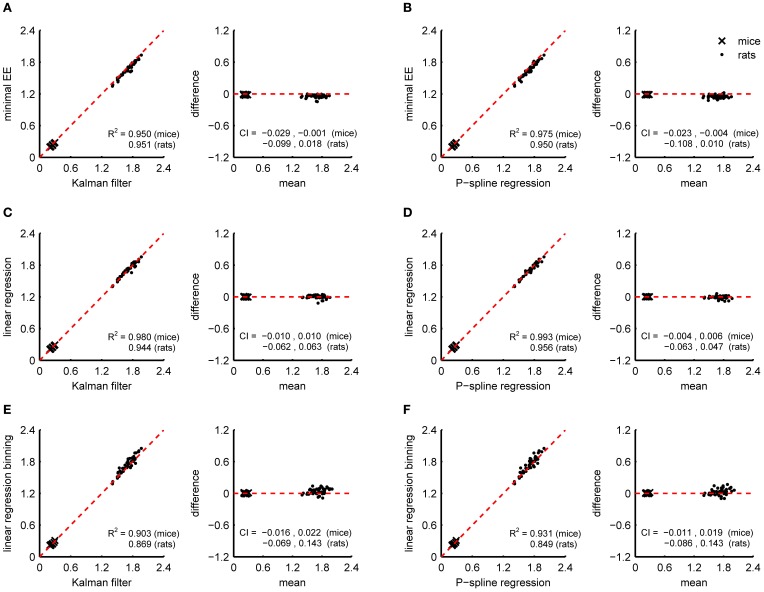
**Correspondence of BMR estimates from indirect calorimetry datasets of mice and rats with the minimal energy expenditure approach, linear regression, Kalman filtering and P-spline regression.** Bland Altman plots are shown of BMR estimates of Kalman filtering and P-spline regression against minimal EE **(A,B)**, linear regression using a linear compartment model to account for gas mixing effects **(C,D)**, and linear regression using binning **(E,F)**. The coefficient of determination (*R*^2^) and 95% confidence interval (CI) are shown separately for mice and rats.

Since the RMR is relatively stable when it has converged to the BMR after a period of fasting, the BMR can also be estimated by methods that assume a constant resting energy expenditure. We compared the BMR estimate of the Kalman filter and P-spline regression with the minimal energy expenditure (Figures 5A,B) and with the BMR estimated by linear regression using the linear compartment model for gas diffusion effects (Figures 5C,D). Both methods were applied on the data obtained during the 90 min period prior to feeding. To reduce the downward bias caused by randomly occurring dips in the RMR, we calculated the minimum averaged energy expenditure for a 5 min window.

Both the minimal EE as the BMR estimate from linear regression had a good correspondence with that of Kalman filtering and P-spline regression (*R*^2^ > 0.94). However, the minimal EE was biased downwards with respect to the BMR estimate of the Kalman filter and P-spline regression, which means that additional postprocessing of the results to correct for the bias is necessary when using this method. In comparison, the BMR estimate by linear regression was not biased and also strongly correlated with the result of the Kalman filter and P-spline regression, suggesting that linear regression is an adequate alternative for estimating the BMR when the RMR has stabilized. Estimating the BMR with linear regression while binning PA and TEE in 15 min intervals gave worse results than the linear compartment model (Figures 5E,F). The larger divergence from the Kalman filter and P-spline regression estimates can be explained both by the larger residual variance obtained by binning (Figure 2C) and by the larger uncertainty that is present in the BMR estimate because regression was based on less data points.

## Influence of experimental variables

The accuracy with which the computational methods described in the previous section are able to partition the TEE into a time sequence of the activity and resting energy component depends on a number of properties and settings of the indirect calorimetry system. Most importantly, these are the sampling frequency with which the respiratory exchange and activity are measured, the type and accuracy of the activity sensor, and the size of the chamber in proportion to the flow rate. Since the estimates of the TEF, BMR, and activity and resting RQ are derived from the result of the decomposition method, it is important to know how the experimental settings affect a method's robustness and, in turn, that of the inferred metabolic measures.

We here tested how the eight metabolic measures presented in the last section are affected by changes in the sampling resolution, the accuracy of the activity sensor, and the system's washout time for the existing experimental data. For each case the effects of a change in the experimental setting was simulated on the existing data, and subsequently component analysis was performed on the new data; for details regarding the simulation procedure, see the respective sections. The estimate of each parameter on the new data was then compared with its basal estimate and used to calculate the mean squared error (MSE) relative to the total variance for the Kalman filter and P-spline regression:

$$\text{relative MSE}(x)=\frac{{\sum_{i}\left(x_{i}-x_{i^{i}}^{\text{basal}}\right)}^{2}}{\sum_{i}{\left(x_{i^{i}}^{\text{basal}}-{\bar{x}}_{i}{}^{\text{basal}}\right)}^{2}}\cdot 100\%$$

with *x*_*i*_ the estimate of metabolic measure *x* for subject *i* on the new data and *x*^basal^_*i*_ the estimate on the original data. Importantly, the relative MSE approximates the unexplained variance 1 – *R*^2^, except that it also penalizes a difference in the mean estimates; this property assists in the interpretation of the relative MSE since it can be directly related to the *R*^2^. It should also be noted that since the relative MSE is auto-referenced (i.e., to the basal estimate) it does not include systemic errors due to drift in the CO_2_ and O_2_ sensors or the mass flow controller.

Since the choice of the filter parameters became sub-optimal when applying the Kalman filter on the data with the simulated experimental changes, we adjusted these parameters as to minimize the total MSE of the Kalman filter. No parameters were changed for the P-spline regression method on the new data.

### Sampling resolution

The sampling frequency with which the respiratory exchange can be measured depends on the type of indirect calorimetry system. For single channel systems a set of O_2_ and CO_2_ analysers continuously measure the respiratory exchange in a single chamber, and therefore there are no restraints on the sampling frequency other than technical limitations related to data storage and data handling. Gas sensors, though, are rather expensive and therefore most commercial manufacturers of indirect calorimetry systems have opted for a multiplexed design where a single set of gas sensors measure the air from multiple chambers in succession. This comes at the cost of reducing the time resolution that can be attained, because now the tubing between each chamber and the sensors needs to be purged every time a new measurement is taken.

Sampling frequency can have a huge impact on the performance of decomposition algorithms and also puts a limit on the amount of detail that can be attained in the estimate of the time-dependent RMR. In fact, recently it has been questioned whether detailed component analysis is even possible on data that has been generated by multichannel systems (Even and Nadkarni, 2012). Hence, it is important to quantify the influence sampling frequency has on the precision with which metabolic parameters are estimated, in order to understand what can be achieved with a certain indirect calorimetry system or experimental design. We therefore performed TEE decomposition on a range of downsampled versions of the original high resolution VO_2_ and VCO_2_ time sequences and compared the estimates of the eight metabolic parameters with their basal estimates. To simulate a dataset with a sample time of *T* seconds we took every *N*-th measurement of the VO_2_ and VCO_2_ while discarding the rest, where *N* is defined as *T* divided by the sample time of the original dataset. Since there are typically no limitations on the sampling frequency with which activity can be measured, we did not perform downsampling of the activity data.

**Figure 6:**
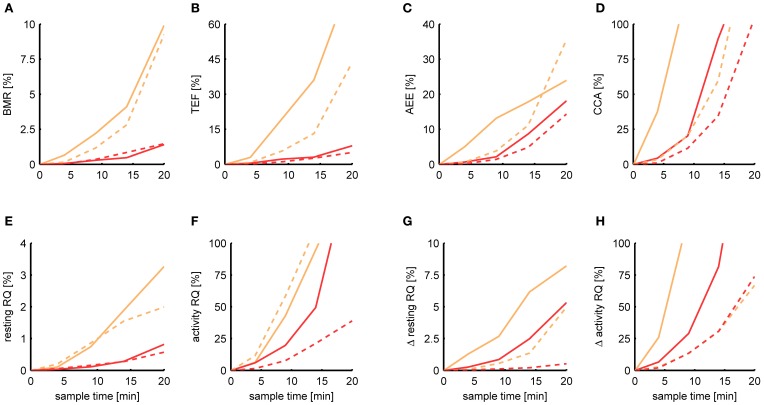
**Dependence of the estimation accuracy on the sample time.** The relative mean squared error is shown of the BMR **(A)**, TEF **(B)**, AEE **(C)**, CCA **(D)**, resting RQ during fasting **(E)**, activity RQ during fasting **(F)**, increase in resting RQ after feeding **(G)**, increase in activity RQ after feeding **(H)** as estimated by the Kalman filter (orange) and P-spline regression (red) on data from mice (solid) and rats (dashed) with a sample time of 4, 9, 14, and 20 min. The relative mean squared error was calculated as the difference of the measure's estimate for a given sample time with the basal estimate at a sample time of 5 s, divided by the basal variance for that measure in mice and rats.

Figure 6 shows the relative MSE of Kalman filtering and P-spline regression in estimating each metabolic parameter for a sample time ranging from 5 s to 20 min. In general, the deviance of each estimated parameter from its basal estimate grows with increasing sample time. The BMR and resting RQ during fasting seem to be the parameters that are most robust against increasing sample time. At a sample time of *T* = 9 min, the MSE of the BMR estimated with P-spline regression is 0.3% for mice and rats, and the MSE of the resting RQ during fasting lies around 0.2%, showing that for these measures there is an exceptionally high correlation of *R*^2^ > 0.997 with the basal estimate for *T* = 9 min. The MSE of the increase in resting RQ after feeding in mice is with 0.9% larger than that of the resting RQ during fasting, but still very robust. Surprisingly, also the AEE and TEF can still be estimated with a decent amount of accuracy at *T* = 9 min: the AEE has an MSE of 2.2% (mice) and 1.4% (rats) and the TEF has an MSE of 2.2% (mice) and 1.1% (rats). In comparison, the other measures are more susceptible to increasing sample times. At *T* = 9 min, the MSE of the CCA is 20.3% (mice) and 11.4% (rats) and the MSE of the activity RQ during fasting is 19.7% (mice) and 7.6% (rats). These errors correspond to an *R*^2^ with the basal estimate in the range of 0.8–0.9, which clearly show that these parameters cannot be measured with a high degree of accuracy in data coming from multichannel systems.

These results show that, in general, the measures calculated from the resting respiratory exchange are less sensitive to decreasing sample rates than those related to activity metabolism. The larger sensitivity of the activity related measures, but also the TEF, is mostly due to the fact that their calculation explicitly depends on the time-dependency of the AEE and RMR estimates, and therefore suffer more heavily from the loss in time resolution. The difference in the robustness between the AEE and CCA is due to the fact that there is a much smaller basal variance in the CCA, which makes the denominator of the relative MSE smaller.

In comparison, the estimates of the metabolic parameters based on the decomposition of the Kalman filter are less robust to lower sample rates, in both mice and rats. We have found the same result in our earlier study, where we compared the performance of both methods on simulated data (Van Klinken et al., 2012). We think that the main cause for the difference in robustness is that, by its design, the Kalman filter estimates the AEE and RMR at a single time point using only a few past measurements whereas the P-spline model takes into account a larger set of local measurements which ensures more stable estimates. In addition, P-spline regression first applies the linear compartment model on the high resolution activity data and then resamples it to the measurement times of the respiratory exchange, which yields a much better correlation between the PA and TEE than when the activity data is first downsampled and then the gas mixing effects are applied, which is what occurs in the Kalman filter.

It is important to note that in our analysis we did not include the effect of additional measurement errors in the O_2_ and CO_2_ data that can occur due to switching between chambers by a multiplexer. When the switching between chambers occurs too fast with respect to the system configuration then old air will remain in the tubes and will affect the new measurement. In practice a choice will have to be made between a higher time resolution on one hand, and therefore more data and a better decomposition, and a higher accuracy of O_2_ and CO_2_ measurements on the other. In our experience the presence of old air can be modeled with an exponential decay curve, which means that the relative error induced by old air can be quantified as a function of the decay rate and the purging time of the system. As an example, for the multiplexed indirect calorimetry system we have currently in our facility the decay rate is approximately 10 s at an excurrent flow rate of 0.4 l/min, which means that with a purging time of 60 s, 99.8% of the old air is purged, yielding a total sample time of 9 min given an 8 cage system and an empty cage for reference air.

### Activity measurements

Several devices exist for quantifying the level of spontaneous PA in rodents, such as photocell sensors (Nonogaki et al., 2003; Bjursell et al., 2008; Kotz et al., 2008), piezo-electric force transducers (Even et al., 1991, 1994), microwave radar systems (Brown et al., 1991; Pasquali et al., 2006) and video-tracking systems (Poirrier et al., 2006). A frequently used approach in commercially available metabolic chamber systems is to measure PA as the number of infrared beam interruptions. It has been suggested, however, that infrared beam interruptions may miss out on the more subtle types of activity (Even and Nadkarni, 2012), which would make this type of sensor less suitable for TEE decomposition. Another disadvantage is that count data is inherently noisy because of the random character of beam interruption occurrences. The electrical signal generated by piezo-elements has been proposed as a more accurate alternative for measuring PA in rodents and has been suggested to correlate more tightly with the AEE and be more sensitive to small movements (Even and Nadkarni, 2012).

Since the datasets used in this study were measured only with piezo-electric force transducers, we were not able to make a direct comparison between this and other types of sensors. We therefore investigated what the effect is of the accuracy of the activity sensor on TEE decomposition in general, by adding noise to the measured activity data. Noise was simulated by multiplying the activity time sequence with the random sequence 1 + *e*_PA_(*t*), where *e*_PA_(*t*) is a slowly varying random process that has been modeled with spline functions. In detail, we took *e*_PA_(*t*) = Σ_*i*_ ε_*i*_ *B*_*i*_(*t*) with *B*_*i*_(*t*) the cubic B-spline basis function with randomly distributed coefficients ε_*i*_ ~ *N*(0, σ^2^) and with 20 knots/h. The reason to model *e*_PA_(*t*) as autocorrelated noise and not as white noise is that in the former case the situation is emulated where each activity bout has a different CCA, which is a reflection of what happens when an activity sensor is less sensitive for detecting certain kinds of activity.

**Figure 7:**
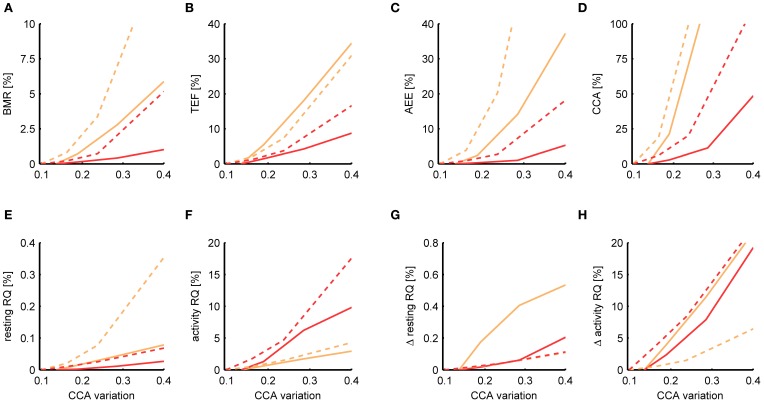
**Dependence of the estimation accuracy on the accuracy of the activity sensor.** The relative mean squared error is shown of the BMR **(A)**, TEF **(B)**, AEE **(C)**, CCA **(D)**, resting RQ during fasting **(E)**, activity RQ during fasting **(F)**, increase in resting RQ after feeding **(G)**, increase in activity RQ after feeding **(H)** as estimated by the Kalman filter (orange) and P-spline regression (red) on data from mice (solid) and rats (dashed) with different levels of simulated noise on the activity data. The relative mean squared error was calculated as the difference of the measure's estimate for a given noise level with the basal estimate with no added noise, divided by the basal variance for that measure in mice and rats. The noise level (*x*-axis) is quantified as the amount of time variation σ_CCA_ in the CCA assuming a multiplicative model CCA(*t*) = CCA_const_· [1 + *e*_CCA_(*t*)] The baseline σ_CCA_ estimates and standard deviation are 0.136 ± 0.025 for mice and 0.099 ± 0.047 for rats.

Figure 7 shows the deviance of the estimated metabolic parameters for various levels of activity noise *e*_PA_(*t*). Since from the result of TEE decomposition it is not possible to discern between noise present in the activity sequence and natural variations in the CCA, we used the amount of variation σ_CCA_ that was present in the CCA estimate as a combined measure of the activity noise. In this way we were able to determine the effect of the simulated noise on top of the basal level of variation in CCA and could make comparisons with the basal σ_CCA_ of other activity sensors. For the time variation in the CCA we assumed a multiplicative model

(5) $$\text{CCA}(t)={\text{CCA}}_{\text{const}}\times \left(1+e_{\text{CCA}}(t)\right)$$

with CCA_const_ and *e*_CCA_(t) the constant and time-varying part of the CCA. The variation σ_CCA_ was estimated using maximum likelihood; for details see Van Klinken et al. (2012), supplemental material 1. The basal CCA variation (mean ± *SD*) was found to be 0.136 ± 0.025 in mice and 0.099 ± 0.047 in rats. In our earlier study we measured respiratory exchange in a mouse at a high resolution using infrared beam sensors to quantify activity (Van Klinken et al., 2012); from this data we estimated σ_CCA_ to be 0.164, which lies within the 95% confidence interval of the σ_CCA_ estimate for piezo-electric sensors. Future research is needed to make a more sound comparison between these activity sensors, performing experiments in which activity is measured simultaneously with both sensors.

From Figure 7 it follows that the estimates of the resting RQ during fasting and the increase after food intake are very robust to activity noise, having a relative MSE of less than 0.2% for all σ_CCA_. Also the BMR was relatively robust, having a MSE of 0.4% (mice) and 0.7% (rats) at a level of twice the basal CCA variation. The AEE estimate was more sensitive to activity noise with an MSE of 1.0% (mice) and 2.7% (rats) at twice the basal CCA variation, and the TEF had an MSE of 4.2% (mice) and 3.7% (rats). The activity RQ during fasting was very sensitive to the activity noise using P-spline regression but not using the Kalman filter, which had an MSE of 1.7% (mice) and 1.5% (rats) at twice the basal σ_CCA_. Investigating this specific observation we found that the additional uncertainty that comes with fitting the activity noise that is part of the P-splines regression algorithm had caused the larger MSE. For the other measures the P-spline regression proved to be more robust to increasing activity noise than the Kalman filter. This result is related to the use of weighed regression, which mitigates the effect of noisy activity data, and also to the choice we made for a relatively low number of knots, which prevented the P-spline model to overfit to the activity noise.

### Chamber washout time

Because of the mixing process of the exhaled air with the air in the metabolic chamber, the time pattern in the O_2_ consumption and CO_2_ production rate measured at the chamber's outlet becomes dampened. In the ideal case, gas mixing acts as a first-order low-pass filter on the instantaneous respiratory exchange, attenuating the high frequency variations that are due to activity. The extent of the dampening effect is directly related to the washout time τ, i.e., to the proportion of the chamber size to the rate of the air flow: all frequencies above the cutoff frequency of *f* = (2πτ)^−1^ are attenuated by the mixing process. Since component analysis ultimately relies on the time correlation between the energy expenditure and activity signal, it is important to know what effect the washout time τ has on the accuracy of the estimated components.

In order to determine the deviance from the basal estimate for varying τ, we deconvoluted the original data for the washout time of the respective experiments, and subsequently re-convoluted the data for a range of different τ's. Since re-convolution with a larger washout time implied that high frequency sensor noise was attenuated, we added white noise such that the power of frequency components above 1 min^−1^ was equal for each derived dataset.

**Figure 8:**
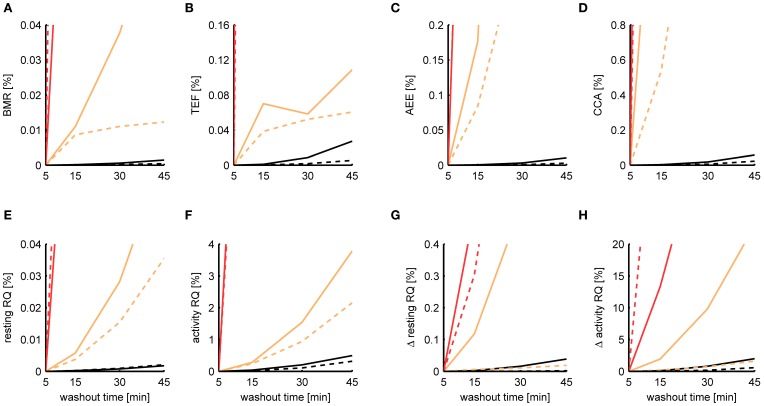
**Dependence of the estimation accuracy on the chamber washout time.** The relative mean squared error is shown of the BMR **(A)**, TEF **(B)**, AEE **(C)**, CCA **(D)**, resting RQ during fasting **(E)**, activity RQ during fasting **(F)**, increase in resting RQ after feeding **(G)**, increase in activity RQ after feeding **(H)** as estimated by the Kalman filter (orange), P-spline regression (red) and P-spline regression using deconvolution (black) on data from mice (solid) and rats (dashed) with simulated chamber washout times of 5, 15, 30, and 45 min. The relative mean squared error was calculated as the difference of the measure's estimate for a given washout time with the basal estimate at a washout time of 5 min, divided by the basal variance for that measure in mice and rats. Graphs focus on showing the difference between the relative MSE of the Kalman filter and P-spline regression with deconvolution; as a result, the MSE of the ordinary P-spline model lies outside the range of the *y*-axis for most washout times. Note that because part of the Kalman filter's MSE of the TEF estimate is composed of a mean difference, the MSE does not increase monotonically for τ.

When initially comparing the results between P-spline regression and the Kalman filter, we found that the estimates of the Kalman filter were much more robust to increasing washout times than those from P-spline regression, having a smaller MSE by a factor of 2–10 depending on the metabolic measure. The reason for the bad performance of the P-spline model is that the decomposition is based on the regression of the TEE on the PA, which becomes increasingly more difficult for larger τ since the correlation between TEE and PA fades away. In contrast, the Kalman filter inherently performs deconvolution, which is the preferred approach when τ is large. We therefore also performed P-spline regression on data where the VO_2_ and VCO_2_ time sequences had been deconvoluted. As shown in Figure 8, the results of P-spline regression on deconvoluted data gave much more accurate results, yielding estimation errors that are only a fraction of those that are obtained when increasing sample times or activity noise. For all measures, except the activity RQ and delta activity RQ, the relative MSE is smaller than 0.1% for the whole range of τ using P-spline regression.

It is important to note that in this experiment we modeled the gas diffusion effects of the larger (simulated) chamber as a linear compartment, which was also the main assumption of the deconvolution process. In this idealized situation deconvolution gives extremely accurate results, because the only source of the estimation error is the amplified sensor noise. In practice, therefore, the performance of P-spline regression and Kalman filtering on data measured with a large washout time will be worse because of deviance from the linear compartment model due to non-ideal mixing. Nevertheless, the key point here is that P-spline regression—and any other type of regression approach for that matter—can only work directly on indirect calorimetry data if the washout time is small, preferably with a chamber volume to air flow ratio of less than 10 min; otherwise the data needs to be deconvoluted first in order to perform robust component analysis.

## Discussion

Component analysis has evolved to become an integral part of indirect calorimetry data analysis, and has proved valuable in studies of obesity to elucidate the interaction of energy expenditure with PA (Girardier et al., 1995; Speakman and Selman, 2003; Novak et al., 2006; Kotz et al., 2008; Maclean et al., 2009; Virtue et al., 2012) and food intake (Maclean et al., 2004; Hambly et al., 2005; Johnston et al., 2007). In addition, component analysis has been used to investigate the influence of body composition on the BMR (Johnson et al., 2001; Selman et al., 2001) and of gene mutations (Mokhtarian et al., 1996; Nonogaki et al., 2003). In order to be able to get quantitative insight into these complex interactions and shed light on the mechanisms of body weight regulation, it is essential that the energy components are estimated with maximum accuracy. In this work we discussed the computational techniques that can be used for component analysis and we dealt with issues regarding data preprocessing and the effect of different experimental settings on the estimation accuracy. To fully test the capabilities of these algorithms and compare their results we have used indirect calorimetry data measured in mice and rats.

Making a basal comparison between a set of metabolic measures that were derived from the results of the Kalman filter and P-spline regression, we found that for the BMR, TEF, AEE and resting RQ there was a high agreement between both methods (*R*^2^ > 0.86), meaning that for these parameters there is virtually no difference in what method is used. For the CCA the correlation was less strong (*R*^2^ = 0.59–0.80), which was mainly caused by the low within group variance for this parameter. In contrast, for the activity RQ in rats large differences were reported between both methods, suggesting that this measure is difficult to estimate reliably, especially if periods of activity are few and short. With the current data it was not possible to determine whether estimates based on the results of either the Kalman filter or P-spline regression were superior. Future research is required to investigate this issue in more depth, for instance by comparing the activity RQ with measures of muscle function.

An important advantage of Kalman filtering and P-spline regression is that they provide time-dependent estimates of the activity and resting VO_2_ and VCO_2_, which makes it possible to assess the dynamic response of energy metabolism on food intake and other metabolic challenges. Other computational approaches, such as ordinary linear regression and minimal energy expenditure estimation, assume that the RMR is constant and can therefore not be used to look at the time variation in the RMR. These methods are tailored to estimate the RMR on relatively short time intervals when the RMR has stabilized, for instance to determine the BMR.

We compared the BMR estimate obtained by linear regression and the minimal energy expenditure with the estimates from Kalman filtering and P-spline regression, and found that there was a strong correlation between these methods (*R*^2^ > 0.94). However, the minimal energy expenditure estimate displayed a downward bias with respect to the other methods, which means that postprocessing of the results of this method is required to correct for this effect. In addition we showed that binning is not as accurate as the linear compartment model to account for the gas mixing effect and to align indirect calorimetry and activity data, because it gives a larger residual error and more uncertainty in the BMR estimate.

Importantly, linear regression in conjunction with binning is not advised for determining the daily AEE and RMR from indirect calorimetry datasets spanning over multiple days, since the resulting estimates will be strongly biased due to the circadian rhythm in the resting energy expenditure. Specifically, the low-pass filter functioning of binning will make it difficult to distinguish between the AEE and the increase in RMR that occurs during the active period of the 24 h cycle, resulting in that the estimate of the daily RMR will approach the RMR of the inactive period of the 24 h cycle, while the AEE will include the increase in RMR during the active period of the 24 h cycle. Therefore, linear regression should always be performed on intervals during which the RMR is stable and by employing a linear compartment model to either convolute the activity data with the impulse response *h(t)* of the chamber or else deconvolute the calorimetry data.

The ability of the Kalman filter and the P-spline regression model to reliably estimate the time dependency in the resting and activity metabolism comes at the cost of an increased complexity of these methods. Most importantly for the user this means that a number of parameters needs to be set in advance, which affect the performance of the method. For the Kalman filter five parameters need to be set, of which the most important ones are the process noise variances associated with the CCA and RMR. These variances control how quickly each process is allowed to fluctuate and determine whether a change in energy expenditure is attributed to (a change in) the CCA, RMR or measurement noise. The P-spline method has comparable parameters, namely the number of knots that is used in the spline function of the RMR and CCA. An additional parameter that we introduced in this study is the amount of weight that is given in the regression to data measured during activity periods. A weight of zero discards data measured during activity bouts, which has the advantage of decreasing the sensitivity to inaccuracies in the activity data, but it also increases the uncertainty in the global estimate because less data points are used. We found that on the data analysed in this study a relative weight for activity periods of 0.2 gave robust estimates.

It went beyond the scope of the present study to investigate more technical issues regarding the sensitivity of both methods to the choice of their parameters. This is an important direction for future research because some metabolic measures, such as the activity RQ, show a large sensitivity to the parameter choice (Table 1). Therefore, more objective criteria need to be found to assist in the standardization of time-dependent component analysis and to eliminate the subjective bias introduced into the estimates of sensitive metabolic measures by the manual tuning of parameters.

The standard for component analysis is to use high time-resolution data, that is, measured with a sample time of 10 s or less, and to use relatively small cages in order to diminish the effect of gas mixing on the time pattern of the respiratory exchange. Many experimental studies, however, are done in multiplexed systems where respiratory exchange is sampled at a much lower rate and activity is measured with infrared beam breaks. This raises the question of whether any useful metabolic parameters can be derived from the datasets generated by these systems (Even and Nadkarni, 2012). We found in the present study that the BMR and resting RQ during fasting were robust measures against an increasing sample time: for a sample time of 9 min the increase in estimation error in these measures with P-spline regression was not more than 0.3% of the within group variance. The Δ resting RQ after feeding and the TEF and AEE were less robust to changes in the sample time but were still estimated with reasonable accuracy, having a relative error of 3% for a sample time of 9 min. In contrast, the CCA and activity RQ showed a very large sensitivity and could therefore not be reproduced at lower sample rates. Overall the estimation error of the Kalman filter was found to be more sensitive to an increasing sample time than P-spline regression, showing that the latter approach is preferred for analysing data from multiplexed systems.

Based on these data it is difficult to say when the additional estimation error introduced by a lower sample rate has become too large to reliably estimate a given parameter. This will depend on the particular experimental study and, more specifically, on whether the larger number of rodents that can be simultaneously measured in multichannel systems and the fact that animals can typically reside longer in such cages can compensate for the increase in estimation error and the reduction in statistical power. A potential solution to increase the sample rate is to perform continuous data acquisition in multiplexed systems, which permits to predict the actual O_2_ and CO_2_ concentrations in the present chamber by extrapolating the transitional O_2_ and CO_2_ concentration of the mixed air by fitting a series of exponential decay curves to the data.

Interestingly, we found that the effect of a reduction in the accuracy of the activity sensor and a larger washout time on the decomposition methods is smaller than the effect of measuring at a low time resolution. In fact, the effect of larger washout times on the estimation accuracy can almost be eliminated, as long as data is measured with a high time-resolution such that deconvolution can be performed. This means that having frequently sampled data is more important for performing robust component analysis than having high accuracy activity sensors or a small washout time.

Concluding, component analysis is an important part of indirect calorimetry data analysis that can provide great additional insight into these datasets. Preferably component analysis is performed on data with a high time-resolution, because this increases the robustness of the decomposition methods, enables the assessment of fast dynamic responses of metabolism on experimental interventions, and permits the calculation of the instantaneous respiratory exchange by means of deconvolution. On low time-resolution data component analysis can be used to measure the BMR or the gradual changes in the RMR associated with circadian rhythm and long term adaptations. The assessment of AEE and TEF can also be feasible in certain cases, but only if the sample time does not exceed 10 min and with the knowledge that the power of subsequent statistical tests may be substantially reduced. On high resolution data from indirect calorimetry systems with continuous data acquisition the Kalman filter and P-spline regression model give very similar results and can therefore both be used. In contrast, for data generated by multichannel system the P-spline regression is advised since it is more robust to infrequently sampled data.

### Conflict of interest statement

Ko Willems van Dijk has a research collaboration agreement with TSE systems (Bad Homburg, Germany), who also provided funding for the development of the penalised spline regression method in the form of indirect calorimetry equipment. There are no patents or marketed products to declare. The other authors declare that the research was conducted in the absence of any commercial or financial relationships that could be construed as a potential conflict of interest.
